# Drought conditions alter litter decomposition and nutrient release of litter types in an agroforestry system of China

**DOI:** 10.1002/ece3.6264

**Published:** 2020-07-16

**Authors:** Tingting Xie, Lishan Shan, Peixi Su

**Affiliations:** ^1^ College of Forestry Gansu Agricultural University Lanzhou China; ^2^ Key Laboratory of Land Surface Process and Climate Change in Cold and Arid Regions Northwest Institute of Eco‐Environment and Resources Chinese Academy of Sciences Lanzhou China

**Keywords:** agroforestry system, litter decomposition, mixture interaction, nutrient dynamics, soil water

## Abstract

Evaluating how decomposition rates and litter nutrient release of different litter types respond to changes in water conditions is crucial for understanding global carbon and nutrient cycling. However, it is unclear how decreasing water affects litter mixture interactions for the maize–poplar system in arid regions. Here, the responses of the litter decomposition process and litter mixture interactions in the agroforestry system to changes in water conditions (control, light drought, and moderate drought) were tested. Moderate drought significantly decreased the decomposition rate for poplar leaf and mixed litters, and decomposition rate was significantly reduced for maize straw litter in light and moderate drought stress. The mass loss rates of maize straw and mixed litters were significantly higher than that of the poplar leaf litter under drought conditions, but there was no significant difference among the three litter types in the control. There was no interaction between mass loss of the mixed litter in the control and light drought conditions, and the litter mixture interaction showed nonadditive synergistic interactions under moderate drought. In terms of nutrient release, there was also no interaction between litter mixture with nitrogen and carbon, but there was antagonistic interaction with potassium release under the light drought condition. Our results demonstrate that drought conditions can lead to decreasing decomposition rate and strong changes in the litter mixture interactions from additive effects to nonadditive synergistic effects in moderate drought. Moreover, light drought changed the mixture interaction from an additive effect to an antagonistic interaction for potassium release.

## Graphical abstract

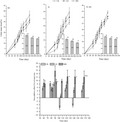

## 1 INTRODUCTION

Litter decomposition plays an important role in the carbon (C) budget and nutrient cycling in terrestrial ecosystems (Bakker, Carreño‐Rocabado, & Poorter, 2011; Gavazov, 2010). Especially in arid and semiarid regions, the ecological roles of litter decomposition include maintenance of ecosystem stability and improvement of soil texture (Wang et al., 2013). In natural terrestrial ecosystems, litter from different species returns to the ground and forms a mixture. Such litter mixtures may change the decomposition rate and nutrient release pattern (De Marco, Meola, Maisto, Giordano, & Santo, 2011; Lecerf et al., 2011). There are two types of effects in the decomposition process of litter mixtures: (a) additive effect (AE), where the decomposition rate of the mixed litter is equal to the average value of the respective component species decomposing alone, which means that there is no interaction among different litter types; (b) nonadditive effect (NAE), where the decomposition rate of mixed litter is higher (synergistic) or lower (antagonistic) than the mean of the single species decomposition because of the chemical and physical changes in leaf mixes (Gartner & Cardon, 2004; Hättenschwiler, Tiunov, & Scheu, 2005), which suggests that interactions among different litter types affect the decomposition process. Gartner and Cardon (2004) reviewed emerging research on mass loss when leaves of different species decayed in mixtures and found nonadditive patterns of mass loss in 67% of tested mixtures. Therefore, understanding these interactions for different litter mixtures is essential, because litter cannot be clearly separated into single species in most ecosystems.

Predicting terrestrial litter decomposition responses to global change has been a major challenge for ecological research in recent years (Incerti et al., 2011; Sanaullah, Cornelia, Charrier, & Chabbi, 2012). In arid terrestrial ecosystems, water availability is the most important environmental constraint for decomposition (Campos, Germino, & Graaf, 2017). Changing soil moisture can alter decomposition and nutrient release of litter mixture, which are key regulators of soil fertility and nutrient cycling in many systems. For example, Santonja, Fernandez, Gauquelin, and Baldy (2015) reported that synergistic interactions increased with time and species diversity in litter mixtures, and drought led to decreasing mass loss rates and more antagonistic interactions in the decomposition of litter mixtures in a Mediterranean forest; this could be explained by increasing plant species richness that enhanced decomposer abundance and activity (García‐Palacios, Shaw, Wall, & Hättenschwiler, 2016; Hector, Beale, Minn, Otway, & Lawton, 2000). Additionally, species with higher water‐holding capacity traits enhanced microclimatic conditions for decomposers (Hättenschwiler et al., 2005; Makkonen, Berg, Logtestijn, Hal, & Aerts, 2012) and thereby promoted the decomposition of their co‐occurring litter species. Makkonen et al. (2012) also reported that higher dissimilarity in water‐holding capacity traits between the component species in a litter mixture increased synergistic effects in litter mixtures under limited moisture conditions. However, increased antagonistic effects were observed under improved moisture conditions. Schuster et al. (2016) found that litter interactions reduced remaining mass by 81% in Belgium and 15% in Germany (averaged across mixtures). Drought negated all synergistic effects and even promoted antagonism in some instances, potentially because the microbial activity was enhanced by the higher temperatures and greater moisture in wetter systems (Zhang, Hui, Luo, & Zhou, 2008). These studies indicated that the mixture interaction of different litter species for decomposition rate may differ among drought conditions in different regions, but there is a lack of knowledge regarding how water influences decomposition rate in litter mixtures of agroforestry systems in arid regions of northwestern China.

Poplar‐based agroforestry systems were reported to stock carbon and hence have the potential to mitigate climate change (Xie, Su, An, Shi, & Zhou, 2017). In northwestern China, poplar is one of the most widely planted trees, because it serves as a windbreak in agricultural fields; however, the soil is very poor in this region. To increase yields in the nutrient‐poor soils, crops are frequently irrigated with large amounts of water and fertilizer applications, which is expensive for local farmers (Wang et al., 2010). Litter from poplar trees and crops provides a source of nutrients and organic matter when it decomposes and could help replenish soil fertility (Gnankambary, Bayala, Malmer, Nyberg, & Hien, 2008). Wang, Chang, Fang, and Tian (2014) reported a positive effect of mixing on the rate of litter decomposition for a poplar–agroforestry system in a humid–subhumid region, and the silt loam soil was more conducive to litter decomposition than the clay loam soil, because the silt loam soil had a slightly higher C to N ratio in the soil organic matter, and lower N and P concentrations; therefore, the decomposer community at that site might be more adapted to utilizing the substrate added to the site to increase the decomposition rate compared with the richer clay loam soil. In arid and semiarid ecosystems, enhanced precipitation or soil water availability can significantly promote aboveground litter decomposition (Campos et al., 2017; de Graaff, Throop, Verburg, Arnone, & Campos, 2014). However, the effect of water changes on litter decomposition rate and nutrient release for mixed forest tree litter and annual crop litter has not been studied in poplar–maize agroforestry systems in arid regions of northwestern China. Therefore, to evaluate the impacts of litter mixtures on the decomposition dynamics and how drought may alter these impacts in a poplar–maize agroforestry system, we tested two hypotheses: (a) The effect of decomposition rate is nonadditive when the two litters were mixed; and (b) drought conditions decrease decomposition rate, and alter decomposition and nutrient release patterns from synergistic to antagonistic effects in mixed litter.

## 2 MATERIALS AND METHODS

### 2.1 Study site

The study was conducted at the desert oasis (39º21′N 100º02′E, 1,400 m a.s.l.) of Linze County, in the center of the Hexi Corridor region of Gansu Province, northwestern China. It has a temperate arid desert climate, with an average annual precipitation of 117 mm and a mean annual evaporative demand of over 2,390 mm. Seventy percent of rainfall occurs between June and September. The average temperature is 7.6°C, while maximum temperatures can reach 39°C and minimum temperatures can reach −27°C. The frost‐free period lasts approximately 165 days (Li, Zhao, & Liu, 2013).

### 2.2 Experimental design

In the desert oasis region, poplar (*Populus gansuensis* C. Wang & H.L. Yang) was planted in the late 1980s as a shelter forest tree; a typical poplar–maize configuration of 1,320 m^2^ was selected in this study. The study site was surrounded by 20‐year‐old poplar trees; the distance between trees was 2 m from east to west and 4 m from south to north (39°20′N, 100°07′E). The soil texture is loamy sand, the nutrient is relatively low, and the field capacity was 23.2%. According to previous research, moderate drought had a field capacity of 50 ± 5% (Zhang et al., 2015), and our previous results showed that the soil water content was around 11.6 ± 1.0% when the field capacity was 50 ± 5% in this region (Xie & Su, 2012). Therefore, three types of water treatments were tested in this study: (a) control (9,200 m^3^ ha^−1^, local irrigation), (b) light drought (irrigation reduced by 15%, 7,800 m^3^ ha^−1^), and (c) moderate drought (irrigation reduced by 30%, 6,400 m^3^ ha^−1^). Irrigation started after maize emergence on 23 May 2016. Calibrated siphons were used to deliver the required amount of water into the hose from the irrigation channel, and a water flow meter was placed at the head of the hose for each block. The water irrigation frequency was eight times throughout the crop growth period, and each irrigation amount was 1,150, 977.5, and 805 m^3^ ha^−1^ for the three water treatments. To eliminate lateral water leakage between blocks, a 3–4 m buffer was implemented.

Because poplar trees are the main shelter forest tree and maize is the main planting crop, poplar leaves (PL), maize straw (MS) (including straw and leaf), and mixed poplar leaves and maize straw (PL–MS) were selected for this study. The maize straw was collected after the maize was harvested in late September 2015. The fresh shedding leaves of poplar were collected from October to November 2015 using litter traps suspended in the poplar–maize system; the leaves were then air‐dried and stored at room temperature. After being air‐dried to constant mass, the poplar leaves and maize straw were cut into 3‐cm‐long fragments and then placed into 20 cm × 20 cm polyethylene litterbags (1 mm mesh size). Each litterbag contained 50 g (dry weight) of either single litter material or a 1:1 paired mixture of two litter types.

**Figure 1:**
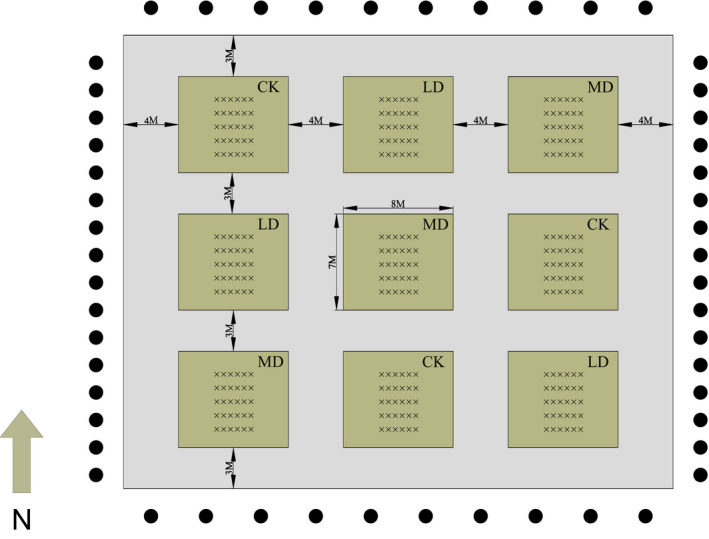
Layout of the experiment design. Control (CK), local irrigation amount (9,200 m^3^ ha^−1^); light drought, 15% reduced irrigation amount (7,800 m^3^ ha^−1^); moderate drought, 30% reduced irrigation amount (6,400 m^3^ ha^−1^)

The experiment was arranged in nested experimental design with three 56‐m^2^ blocks (replicates) for each water treatment (Figure 1). On 1 April 2016, seven litterbags of each type (21 litterbags) were fixed to the ground surface in each block with small pieces of wire (Figure 1). To determine the ratio between the air‐dried mass and oven‐dried mass, six subsamples of litter for each type were oven‐dried at 70°C for 48 hr at the time of initial deployment. This was used to convert the initial air‐dried mass of litter into oven‐dried mass.

### 2.3 Mass loss and chemical analysis

Because the irrigation was stopped when the maize was harvested, one litterbag for each litter type was only retrieved from each block during the crop growth stage at days 60, 80, 90, 105, 120, 140, and 164 after the litter bags were assigned. A total of 189 litterbags (3 litter types × 3 water conditions × 7 sampling dates × 3 replicates) were thus used during the experiment. In the laboratory, extraneous matter such as other plant materials and small animals were removed from the litterbags by hand. The remaining litter was then oven‐dried at 70°C for 48 hr to assess the dry mass.

Chemical composition (carbon, C; nitrogen, N; phosphorus, P; and potassium, K) was analyzed for initial litter and litter during the decomposition process at 105, 140, and 164 days. After determining the remaining dry mass from each block, each litter type was ground and passed through a size 100 mesh screen for chemical analysis. Total N concentrations were determined using the Kjeldahl method. Total P concentrations were determined using the molybdenum blue colorimetric method. Total C concentrations were determined using the dichromate oxidation method. Total K concentrations were determined using flame photometry (Bao, 2000).

### 2.4 Soil water content and soil temperature

Soil water content at a soil depth of 5 cm was also measured at 60, 80, 90, 105, 120, 140, and 164 days by the gravimetric method (Erteka & Kara, 2013). Soil temperature was recorded by WET sensor (Delta‐T Device, Cambridge, UK) at the same time in each block.

### 2.5 Statistical analyses

The decomposition rate (*k*) of litter dry mass was assessed during the experimental periods for each water treatment. The value of the decay constant (*k*) was determined by fitting a simple negative exponential model (Swift, Heal, & Anderson, 1979) as follows:

$$y=e^{-kt}$$

where *y* is the percent of litter dry mass remaining in litterbags at time *t* (month), *e* is the base of the natural logarithm, *k* is the decomposition rate, and *t* is the time.

The expected mass loss and nutrient release of the litter mixture were calculated as the mean mass loss and nutrient release of the litter in the single‐species litterbags in the corresponding control or drought treatments. Then, the NAE was calculated as follows:

$$\text{NAE}=\frac{\left(O-P\right)}{P}\times 100$$

where O is the observed value for each replicate, and P is the predicted value. We used paired *t* tests for the litter mixture to test whether NAE was significantly different from zero in the three water treatments. The litter mixture interactions could be additive (no significant difference between observed and predicted values), nonadditive synergistic (higher observed than predicted values), or nonadditive antagonistic (lower observed than predicted values) (Wardle, Bonner, & Nicholson, 1997).

A paired *t* test was used to compare initial litter chemistry of the two plants with a significance level of *p* < .05. We performed a four‐way ANOVA to test the effects of water, litter types, time, and block on litter mass loss and nutrient release. Tukey's test was used for multiple comparisons among treatments. Additionally, three‐way ANOVA was performed to test the effect of water, time, and block on soil water content and soil temperature, and the effect of water, litter type, and block on the decomposition rates (*k*‐value). Data analyses were performed using SPSS Statistics 13.0, and figures were drawn by Origin 8.

## 3 RESULTS

### 3.1 Soil microclimate condition

**Figure 2:**
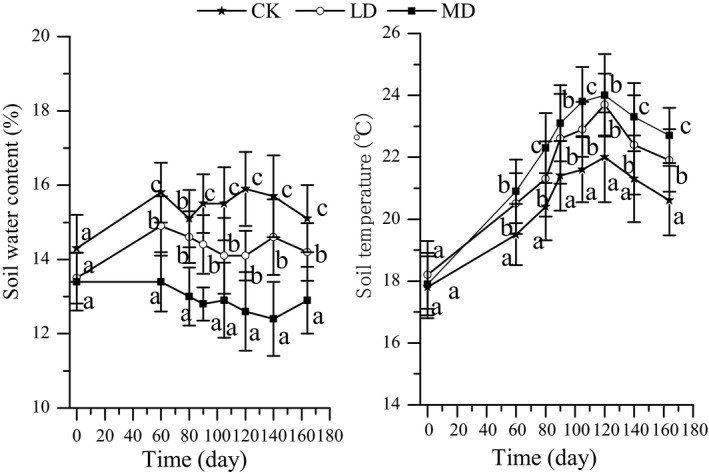
The soil water content and soil temperature during the decomposition process in drought conditions. Values (mean ± *SE*) with different letters are significantly different (*p* < .05)

The soil water content was highest (average value of 15.4%) in the control and lowest (average value of 12.9%) under moderate drought; there were significant differences among the three water conditions at each sampling time (*p* < .05; Figure 2). The soil temperature was highest (22.3°C) under moderate drought, and drought effect was significant; the light drought stress was 6.31% greater than that of the control, and the moderate drought stress was 10.19% greater than that of the control (*p* < .05; Figure 2).

### 3.2 Initial litter chemistry

The initial concentrations of N (14.4 mg/g), C (448.9 mg/g), K (15.4 mg/g), and P (2.13 mg/g) were significantly lower for poplar leaf than maize straw (N, 17.8 mg/g; C, 475.7 mg/g; K, 21.2 mg/g; and P, 3.47 mg/g), but the initial C/N and C/P ratios for poplar leaf were 16.65% and 72.79% greater than those for maize straw (*p* < .05; Table 1).

**Table 1:** Initial nutrient concentrations in litters of three litter types

Litter	N (mg/g)	C (mg/g)	K (mg/g)	P (mg/g)	C/N	C/P
Poplar leaf	14.4 ± 0.2b	448.9 ± 1.56b	15.4 ± 1.0b	2.13 ± 0.23b	31.17 ± 2.03a	210.75 ± 2.83a
Maize stalk	17.8 ± 0.2a	475.7 ± 1.31a	21.2 ± 1.65a	3.47 ± 0.6a	26.72 ± 1.23b	121.97 ± 1.45b

Note

Different lowercase letters in each column indicate significant differences of three litter types (*p* < .05).

### 3.3 Mass loss and decomposition rate

**Figure 3:**
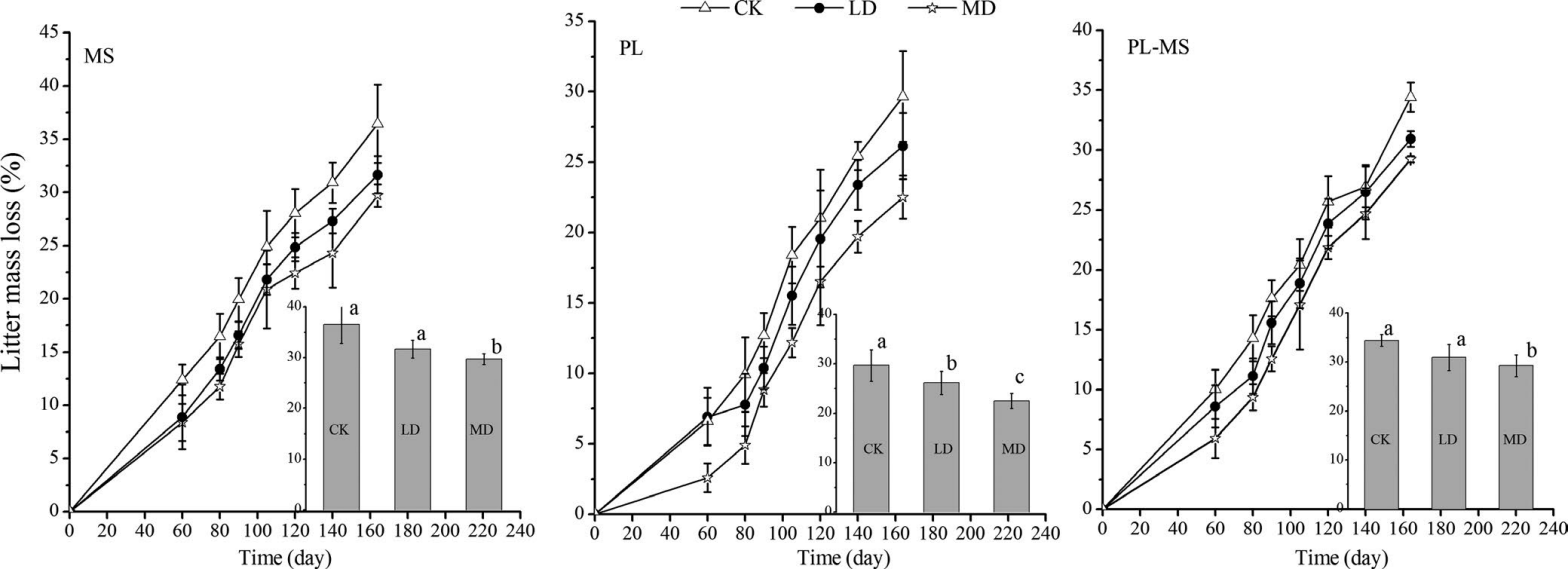
Mass loss of three litter types for different water conditions and mass loss of three water conditions for different litter types. Inset panel shows the effect of different irrigation amount on mass remaining (%) for every litter type or the effect of three litter types on mass loss (%) for every water condition. Values with different letters are significantly different among different water conditions or litter types. MS, maize straw; PL, poplar leaf; PL‐MS, mixed poplar leaf and maize straw; CK, control; LD, light drought; MD, moderate drought

When compared with the initial mass, the maize straw litter lost 29.71%–36.45% of its mass (Figure 3), poplar leaf litter lost 22.51%–29.66% of its mass (Figure 3), and mixed poplar leaf and maize straw litter lost 29.25%–34.41% of its mass (Figure 3) following 164 days of decomposition under different water conditions. Drought effect was significant. For the poplar leaf litter, there were significant differences during the last sampling time among the three water treatments. The mass loss in the control for maize straw litter was 22.68% greater than that for moderate drought, and mass loss under light drought was 6.56% higher than that for moderate drought. The mass loss in the control for mixed litter was 16.58% greater than that for moderate drought, and mass loss under light drought was 5.71% greater than that for moderate drought (*p* < .05; Figure 3).

For the three litter types, the mass loss was highest in maize straw and lowest in poplar leaf. Litter type effect was also significant; mass loss of the maize straw litter was 22.89% greater than that of the poplar leaf litter in the control and was 21.08% and 40.60% greater than that of the poplar leaf litter in light and moderate drought, respectively. The mass losses of mixed litter were 18.29% and 29.94% greater than those for poplar leaf litter in light and moderate drought, respectively (*p* < .05; Figure 3). There was no significant effect on mass loss of the interactions between water and litter type, water and time, time and water, and the three‐way interaction (Table 2).

**Table 2:** Results of 4‐way ANOVAs to test the effects of water condition (*W*), litter types and time (*T*) and block on litter mass loss

Treatments	*df*	*F*‐value	*p*‐value
water condition (*W*)	2	378.85	<.0001
Time (*T*)	6	1743.88	<.0001
litter types (*L*)	2	587.12	<.0001
*W* × *T*	12	1.68	.999
*W* × *L*	4	5.63	.630
*T* × *L*	12	4.54	.897
*W* × *T* × *L*	24	1.57	1.000
Block	8	107.86	.153

Decomposition rates (*k*) differed among treatments (Table 3). All exponential curves were significant (*p* < .05), with *R* ^2^ values ranging from 0.8902 to 0.9735. Drought effect was also significant. For maize straw, the decomposition rate of the control treatment was 14.86% higher than that under light drought, and 26.87% greater than that under moderate drought (*p* < .05); for poplar leaf, that of the control treatment was 30.77% greater than that of moderate drought; and for mixed litter, that of the control treatment was 14.93% greater than that of moderate drought (*p* < .05). For the three litter types, the decomposition rate for the maize straw litter and mixed litter was significantly higher than for the poplar leaf litter in all water treatments (*p* < .05).

**Table 3:** Decomposition rates (*k*, day^−1^) and the associated *R* ^2^ from regression equations of three litter types at different water conditions

Water treatments	Litter types					
	Poplar leaf		Maize stalk		The mixture of poplar leaf + maize stalk	
	*k*‐value (month^−1^)	*R^2^*	*k*‐value(month^−1^)	*R^2^*	*k*‐value(month^−1^)	*R^2^*
CK	0.068 ± 0.006a ^B^	0.8902	0.085 ± 0.005a ^A^	0.9869	0.077 ± 0.005a ^A^	0.9735
LD	0.06 ± 0.003ab^B^	0.9267	0.074 ± 0.004b^A^	0.9728	0.071 ± 0.004ab^A^	0.9613
MD	0.052 ± 0.003b^B^	0.9443	0.067 ± 0.003b ^A^	0.9685	0.067 ± 0.003b^A^	0.9285

Note

Different lowercase letters in each column indicate significant differences of *k*‐values among different water conditions at same litter type, and different superscripted uppercase letters in each row indicate significant differences of *k*‐values among different litter types for the same water condition (*p* < .05).

### 3.4 Litter mixture interaction on mass loss

**Figure 4:**
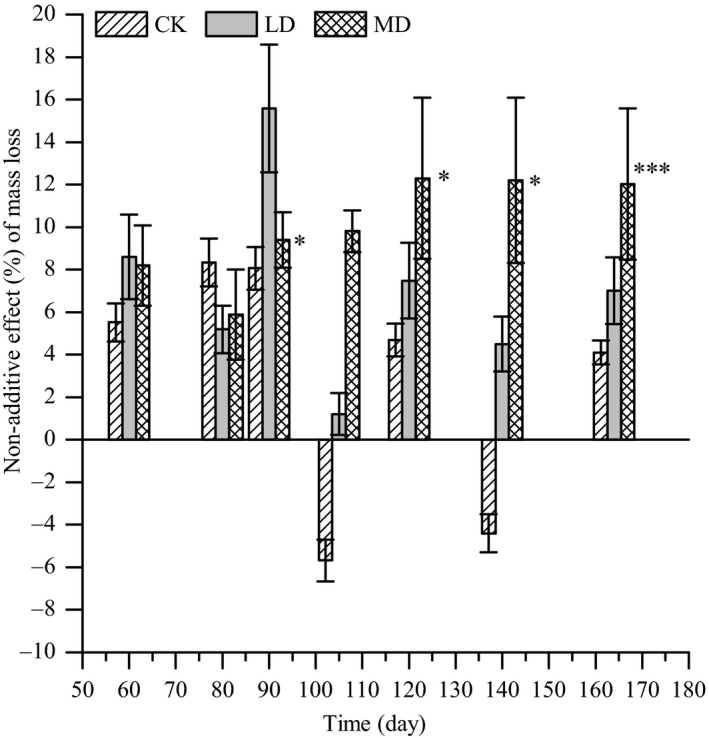
Nonadditive effects (NAE) on litter mass loss of litter mixture for the three water conditions (mean ± *SE*). NAE significantly different from 0 are indicated with symbols: * for *p* < .05 and *** for *p* < .001

The interaction of mixed litter on mass loss varied according to time and water conditions (Figure 4). During the experimental period, NAE only occurred under moderate drought after 90, 120, 140, and 164 days of decomposition and presented as a synergistic interaction. For the control and light drought, AE was seen for the mixed litter interactions at 60, 80, 90, 105, 120, 140, and 164 days.

### 3.5 Nutrient release and litter mixture interaction

**Figure 5:**
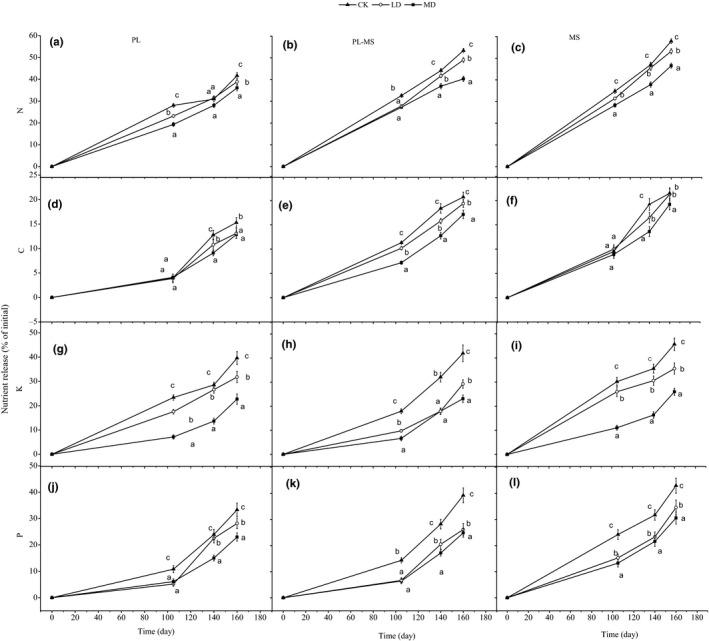
Effects of drought on the release of nitrogen (N), carbon (C), phosphorus (P), and potassium (K) in three litter types during the decomposition period. Values (mean ± SE) with different letters are significantly different (*p* < .05). MS, maize straw; PL, poplar leaf; PL‐MS, mixed poplar leaf and maize straw; CK, control; LD, light drought; MD, moderate drought

**Figure 6:**
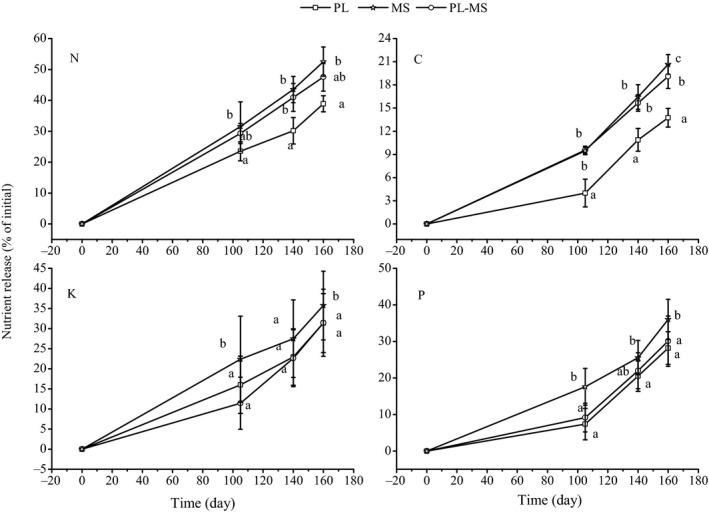
The release of nitrogen (N), carbon (C), phosphorus (P), and potassium (K) of three litter types during the decomposition period. Each data point represents the average over all treatments. Values (mean ± *SE*) with different letters are significantly different (*p* < .05). MS, maize straw; PL, poplar leaf; PL‐MS, mixed poplar leaf and maize straw

When compared with the initial concentrations, N, C, K, and P release increased as decomposition progressed for both single and mixed litter (Figure 5). The increase in N release was higher than that for C, K, and P. For the different water conditions, single and mixed litter N, C, K, and P release decreased with decreasing irrigation following the decomposition period (Figure 5a–l). Drought effect was significant; the N release under light drought was 6.67%–8.21% less than that of the control treatment, and under moderate drought was 13.44%–24.41% less than that of the control treatment after 164 days of decomposition (*p* < .05; Figure 6). The C release under light drought was 1.11%–14.38% less than that of the control treatment, and under moderate drought was 10.60%–17.27% less than that of the control treatment. The K release under light drought was 19.67%–30.23% less than that of the control treatment, and under moderate drought was 42.62%–44.95% less than that of the control treatment. The P release under light drought was 15.48%–33.34% less than that of the control treatment, and under moderate drought was 28.85%–36.60% less than that of the control treatment (*p* < .05; Figure 6).

For the different litter types, the nutrient release for maize straw after the crop growth period of decomposition was higher than that for poplar leaf, but the mixed litter was intermediate. Litter type effect was significant; the N release of maize straw litter was 35.10% greater than that of the poplar leaf litter after 164 days of decomposition (*p* < .05; Figure 6), whereas the C release of maize straw litter was 50.25% greater than that of the poplar leaf litter, and C release of the mixed litter was 38.84% greater than that of the poplar leaf litter (*p* < .05; Figure 6). The P and K releases were 13.86% and 27.87% higher for maize straw litter than poplar leaf litter (*p* < .05; Figure 6). Water, litter type, time, and their interactions all significantly affected N, C, P, and K release, except for the interactive effects of the three factors on P release (*p* < .0001 or *p* < .001; Table 4).

**Table 4:** Results of 4‐way ANOVAs to test the effects of water condition (*W*), litter types and time (*T*) and block on N, C, K, and P (% of initial concentration)

Treatments	*df*	N		C		K		P	
		*F*‐value	P‐value	*F*‐value	P‐value	*F*‐value	P‐value	*F*‐value	P‐value
water condition (*W*)	2	250.54	<.0001	64.99	<.0001	1503.27	<.0001	734.47	<.0001
Time (*T*)	2	1,375.70	<.0001	717.21	<.0001	1,417.43	<.0001	2,732.57	<.0001
litter types (*L*)	2	596.91	<.0001	286.36	<.0001	263.39	<.0001	442.11	<.0001
*W* × *T*	4	9.3	<.0001	6.97	<.0001	8.91	<.0001	17.02	<.01
*W* × *L*	4	4.74	<.01	4.45	<.0001	52.67	<.0001	19.51	<.01
*T* × *L*	4	17.17	<.0001	2.28	<.0001	25.82	<.0001	18.95	<.01
*W* × *T* × *L*	8	5.21	<.0001	1.04	<.0001	8.69	<.0001	6.10	.12
Block	8	104.96	.366	0.59	.785	7.28	<.0001	2.25	.33

**Figure 7:**
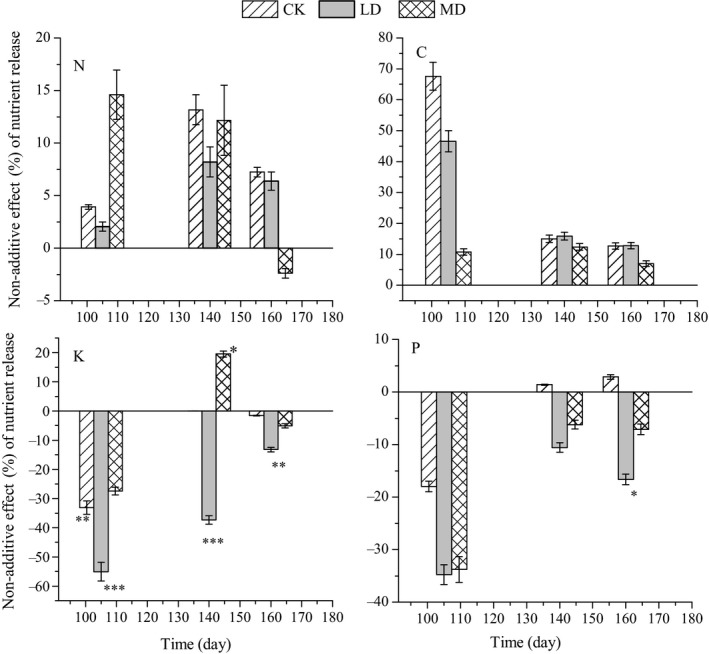
Nonadditive effects (NAE) on nutrient release of litter mixture in the three water conditions (mean ± *SE*). NAE significantly different from 0 are indicated with symbols: * for *p* < .05, ** for *p* < .01, and *** for *p* < .001

The interaction effect of mixed litter on nutrient release was different for different nutrients over time and water conditions (Figure 7). During the experimental period, AE for N and C release was seen for the mixed litter interactions at 105, 140, and 164 days under the three water conditions. There was an NAE on K release under light drought at the three times that presented as an antagonistic interaction. Additionally, the mixture interaction changed no interaction to a synergistic interaction under moderate drought at 105 to 140 days. For P release, an antagonistic interaction was only found under light drought after 160 days of decomposition.

## 4 DISCUSSION

### 4.1 Effect of litter mixture on mass loss and nutrient release

Litter mass loss is controlled by litter quality, which includes N concentration, the C:N ratio, and other chemical properties (Bontti et al., 2009; Hättenschwiler & Jørgensen, 2010; Waring, 2012). Because of the significant differences in initial litter nutrient concentrations, the litter mass and decomposition rate (*k*) between maize straw and poplar leaf litter were significantly different. However, when the litter of the two species was mixed, the litter mass and decomposition rate were not significantly enhanced under control conditions; this is inconsistent with the results from Liu, Huang, Sun, and Han (2010). Previous studies on the effect of mixed litter on mass loss reported that nonadditive interactions (50% synergistic and 20% antagonistic) were prevalent (Gartner & Cardon, 2004). Santonja et al. (2015) found synergistic effects in 64% of cases and no interactions in 36% of cases. Through comparison of the expected and observed mass loss in the litter mixture in the control treatment, we found that the effect of the two species mixed litter on mass loss was no interaction (Figure 4); this is in disagreement with our first hypothesis that the decomposition rate of litter mixture deviates from that of the two species decaying alone. This lack of interaction could be explained by the resource similarity, because the two species in the mixture litter are nutrient‐rich (Table 1), and have similar soil microbial activity in the same soil type (the data are shown in other paper which has not been published) (Chapman & Newman, 2010).

Different decomposed litters, at the same site, differed in their nutrient accumulation and release. When the initial N concentration is low in the litter, soil N often gets immobilized and that affects the soil N dynamics (Li, Yu, Li, Chen, & Liang, 2007). Our results found all nutrients underwent release from the two litters and released nutrients that are incorporated into the soil will be reused by plants in the following few years (Bradford et al., 2002). For different litter types, the nutrient release from litters that had a higher initial nutrient amount was greater than that from litters that had poor nutrients and a higher capacity for nutrient enrichment (Ball, Bradofrd, & Hunter, 2009). We determined that the nutrient release of maize straw litter was significantly higher than that of poplar leaf litter because of the higher initial nutrient content. Moreover, we found that the mixed effects on N, C, P, and K release had no interactions in the control treatment, except for a synergistic NAE for K release in the early decomposition period; this was similar to the mass loss for mixed litter. One potential difference for this finding is that there was a larger difference of initial K concentrations between maize straw and poplar leaf litter (Vos, Ruijven, Berg, Peeters, & Berendse, 2013); in this study, the maize straw had a higher K concentration, which promoted the release of poplar leaves when they were mixed together.

### 4.2 Effect of soil water on decomposition rate and litter mixture interactions

Previous studies on litter decomposition showed that heavy rainfall or increased precipitation decreased (Schuster, 2016; Walter et al., 2013), increased (Anaya, Jaramillo, Martínez‐Yrízar, & García‐Oliva, 2012; Campos et al., 2017), or had no significant effect on the decomposition rate (Zhao, Huang, Ma, Li, & Zhou, 2012). We found that mass loss and nutrient release for all three litter types decreased in drought stress. This result supports our second hypothesis (that drought decreases decomposition rate) and is consistent with previous studies demonstrating low decomposition rates under dry conditions (Andresen et al., 2010; van Meeteren, Tietema, Loon, & Verstraten, 2008; Sanaullah et al., 2012; Wang et al., 2017). However, Haugwitz, Michelsen, and Priemé (2016) found that a presummer drought treatment had a positive impact on litter decomposition, which might be related to higher carbon dioxide respiration and fungal abundance. In our study, decreasing mass loss and decomposition in drought stress might be related to the low irrigation amount. The soil water content also significantly decreased and the soil temperature significantly increased under low irrigation conditions (Figure 2); this condition decreased the soil activity (unpublished data), which supports the theory that moisture is essential in regulating micro‐decomposer activity (García‐Palacios et al., 2016).

In contrast to the findings of Santonja et al. (2015), dry climatic conditions diminish synergistic litter interactions in mixed litter. Over the experiment period, the litter mixture interaction changed from an AE to nonadditive synergistic interactions for mass loss in moderate drought stress, this is in disagreement with our second hypothesis that drought alters decomposition rate by changing from synergistic to antagonistic effects in mixed litter. One of the potential reasons may be that there is dissimilarity in water retention between the two litter species that would improve the microclimate conditions when water is a limiting factor under moderate drought treatment, which thus facilitated positive NAE (Makkonen et al., 2012). If the drought was severe, the synergistic effects on decomposition may be reduced, and this requires further research. For nutrient release, the NAE was only found for K release; the NAE changed from an antagonistic interaction to a synergistic interaction with increasing drought stress over the 140 decomposition days, and it changed from an antagonistic interaction to an AE with increasing drought stress over the 164 decomposition days. This finding is also in disagreement with our second hypothesis that drought alters nutrient release from synergistic to antagonistic effects in mixed litter. Our findings demonstrated that drought stress may have a more severe immediate impact on nutrient cycling than previously thought as a result of stifled litter interactions (Liao, Hou, & Wang, 2002; Walter et al., 2013).

## 5 CONCLUSIONS

Our results highlighted that drought conditions significantly decreased litter mass loss and nutrient release for all litter types. For the mixed litter, the nonadditive synergistic interaction for mass loss was only found under moderate drought, whereas AE was observed in the control and under light drought. For N and C release, the litter mixture interaction presented as AE. NAE of the mixture interactions presented for K release under light drought and presented as an antagonistic interaction; the mixture interaction changed from no interaction to a synergistic interaction under moderate drought from 105 to 140 days. For P release, an antagonistic interaction was only found under light drought after 160 days of decomposition. However, this experiment was only conducted during the crop growth period; therefore, future studies should evaluate if the interactions are maintained for the duration of litter decomposition.

## CONFLICT OF INTEREST

None declared.

## AUTHOR CONTRIBUTION

**Tingitng Xie**: Data curation (lead); Writing‐original draft (lead); Writing‐review & editing (lead). **Lishan Shan**: Investigation (equal); Methodology (equal). **peixi su**: Resources (equal).

## AUTHOR CONTRIBUTIONS

P.X.S: Conceptualization (study); T.T.X. and L.S.S.: Collect and analysis (data). All authors contributed significantly to writing, reviewing, and editing the paper and approved of it for publication.

## Data Availability

Data underlying this article can be accessed on Dryad, and the DOI is https://doi.org/10.5061/dryad.931zcrjgd.
